# Efficiency of Glyphosate to Control Crabgrass in Different Phenological Stages and Soil Water Potentials

**DOI:** 10.3390/plants15010111

**Published:** 2025-12-31

**Authors:** Ricardo Fagundes Marques, Antonio Carlos Silva Junior, Francielly Rodrigues Gomes, Cibele Chalita Martins, Dagoberto Martins

**Affiliations:** Department of Plant Production, School of Agricultural and Veterinary Sciences, São Paulo State University, Jaboticabal 14884-900, Brazil; acsjr_agro@hotmail.com (A.C.S.J.); cibele.chalita@unesp.br (C.C.M.); dagoberto.martins@unesp.br (D.M.)

**Keywords:** *Digitaria nuda*, herbicide, monocotyledons, water deficit, weed

## Abstract

Herbicide efficacy on weeds under water deficit conditions may be reduced because water stress decreases cuticle hydration, thereby limiting the penetration of herbicides such as glyphosate. This study evaluated the efficiency of the herbicide glyphosate to control crabgrass (*Digitaria nuda* Schumach.) in different phenological stages of development and when submitted to distinct soil water potentials. A completely randomized design with four replicates was adopted. The treatments were arranged in a 3 × 3 × 2 factorial scheme, consisting of three soil water conditions (−0.03, −0.07, and −1.5 MPa), three glyphosate doses (0.0, 270.0, and 540.0 g a.e. ha^−1^, corresponding to 0, 50, and 100% of the label-recommended dose, respectively), and two phenological stages (4–6 leaves and 1–3 tillers). The following were evaluated: the specific leaf area, stomatal conductance, the difference between environmental and leaf temperature, and shoot and root dry matter. There is a decrease in crabgrass morphophysiological components according to the increase of water restriction, regardless of the phenological stage evaluated. The 4–6 leaves stage of crabgrass plants influences the control provided by the herbicide glyphosate, irrespective of the dose used. The different water deficits studied reduced the efficiency of the herbicide glyphosate in the two development stages of crabgrass plants.

## 1. Introduction

*Digitaria nuda* Schumach., commonly known as crabgrass, is an annual weed species widely distributed across agricultural regions and considered a potential threat to agroecosystems in at least 60 countries. Its global relevance is associated with high persistence and difficulty of management, even under suppressive conditions such as sugarcane straw cover. Although crop residues can significantly reduce seedling emergence, crabgrass is capable of re-establishing from the soil seedbank, with reported emergence reductions ranging from 62% to 100% depending on residue levels, highlighting its adaptive capacity and persistence in production systems [1].

Chemical control remains one of the primary strategies for managing crabgrass, with glyphosate being widely used for post-emergence applications. Glyphosate is mainly absorbed through leaf tissues and translocated via the symplast to its site of action, where it inhibits the enzyme 5-enolpyruvylshikimate-3-phosphate synthase (EPSPS), disrupting the shikimate pathway and ultimately leading to plant death. The effectiveness of this herbicide, however, is influenced by plant physiological status and environmental conditions at the time of application [2,3].

Under scenarios of climate variability and agricultural expansion into regions characterized by prolonged periods of water deficit, weed species have developed morphological and physiological adaptations aimed at reducing transpirational water loss. These adaptations include stomatal closure, a reduction in leaf area, and modifications of the leaf surface, which collectively contribute to improved tolerance to drought conditions. Such traits may indirectly interfere with herbicide performance by limiting foliar absorption and translocation [4,5].

The alterations in cuticular characteristics associated with drought tolerance, such as increased wax deposition, represent a significant challenge to chemical weed management, as thicker or more complex cuticles can restrict the penetration of foliar-applied herbicides [6]. Consequently, the interaction between plant water status and herbicide efficacy has become an important factor to be considered in weed management programs, particularly in environments subject to water stress.

A comprehensive understanding of soil water availability and its influence on herbicide action is therefore essential to optimize weed control across different developmental stages. Previous studies have demonstrated that reduced soil water potential can negatively affect glyphosate efficiency, reinforcing the need to consider soil moisture conditions when defining management strategies [5]. In this context, the present study evaluated the control efficiency of glyphosate applied to crabgrass at different phenological stages under varying soil water potentials.

## 2. Material and Methods

### 2.1. Experimental Area

This study was installed and conducted at greenhouse conditions located at the Department of Vegetal Production of the Faculty of Agricultural and Veterinary Sciences of São Paulo State University—UNESP, in Jaboticabal, SP, Brazil. The geographic coordinates are 21°14′43.42″ S, and 48°17′32.80″ W Gr., with an altitude of 583 m. During the experiment period, the average temperature registered inside the greenhouse was 26 ± 5 °C, and the relative air humidity was 82 ± 10%.

### 2.2. Experimental Design and Treatment

The experimental design used was completely randomized, with four replicates. The treatments were arranged in a 3 × 3 × 2 factorial scheme, consisting of three soil water conditions (−0.03, −0.07, and −1.5 MPa), three glyphosate doses (0.0, 270.0, and 540.0 g a.e. ha^−1^, corresponding to 0, 50, and 100% of the label-recommended dose, respectively), and two phenological stages (4–6 leaves and 1–3 tillers).The glyphosate doses studied were established depending on the dose of 1.5 L ha^−1^ recommended in the package insert of the commercial product ROUNDUP ORIGINAL^®^ (N-(phosphonomethyl) glycine) (360 g a.e. L^−1^) (Monsanto Company, Saint Louis, MO, USA) for monocotyledonous weed control, which are 0, 50, and 100%.

Each experimental unit was characterized by a polyethylene pot with a volumetric capacity of 2.5 L, fully filled with soil classified as Oxisol. Samples of this soil were collected and sent to laboratory analysis. The chemical and physical characteristics were a pH in CaCl_2_ of 4.9; 10.0 g dm^−3^ of organic matter; a P resin of 4.0 mg dm^−3^; a base saturation of 29.0%; contents of K, Ca, Mg, and H + Al of 0.3, 2.0, 3.0, and 22.0 millimole of charge per cubic decimeter (mmol_c._ dm^−3^), respectively; 77.5% sand; 3.6% silt; and 18.9% clay. Based on chemical analysis, fertilization and soil correction were made using 2.5 g of a formulated fertilizer 04-14-08 and 2.0 g of dolomitic limestone for each kilo of soil.

To ensure uniform soil bulk density and, consequently, comparable soil water retention among all experimental units, the soil was air-dried, sieved, and thoroughly homogenized prior to pot filling. A fixed mass of soil was added to each polyethylene pot and distributed evenly to completely fill the 2.5 L volume, resulting in similar bulk density across all treatments. The soil was gently compacted using a standardized procedure applied consistently to all the pots, minimizing variability in soil structure. This methodology ensured that the differences in soil water potential among the treatments were due to controlled water availability rather than variations in soil physical properties.

### 2.3. Measurements and Sampling

To obtain the soil water retention curve, the Richards pressure plate was used, in which three minimum soil water potentials (Ψs) were established: −0.03, −0.07, and −1.5 MPa (13, 10, and 8%, respectively). The value that represents the maximum capacity of water retention in this soil corresponds to −0.01 MPa. The other potentials (−0.03, −0.07, and −1.5 MPa) are the types of water management used as treatments. The relation of water potentials with their respective percentage of soil humidity is described in Table 1.

The pots were weighed daily, and when their weight reached the soil water potential established for each treatment (−0.03, −0.07, or −1.5 MPa), water lost through evapotranspiration was replenished to restore the soil to its maximum water-holding capacity (14%). A Class A evaporation pan was used to monitor daily evaporation in order to characterize the environmental conditions under which the plants were maintained, since this study was conducted in a greenhouse. The device consists of a circular evaporimeter with a diameter of 1.21 m and a height of 0.254 m, constructed of a 22-gauge galvanized steel sheet, installed on the ground over a wooden support, leveled on the soil surface, and filled with clean water to 5 cm below the upper rim. Water level readings were taken daily, and the difference between successive measurements was used to determine evaporation during the period, as described by [7]. Evaporation data from the Class A pan were subsequently converted to reference evapotranspiration (ETo) (Equation (1)):

(1) ETo = ECA × Kp 

The seeding of the *D. nuda* was performed with a density of 10 seeds per pot. After emergence, before the full development of the first leaf, paring was conducted, leaving only one plant per pot. The soil was maintained with sufficient moisture until the development stage of two fully expanded leaves. After this, the water management of each treatment associated with the doses of glyphosate and the different phenological stages of development of the plants was conducted, which remained until the end of this study.

The different doses of glyphosate were applied once when the plants reached the predefined development stage (4–6 leaves and 1–3 tillers). For the herbicide application, a pressurized sprayer with CO_2_ was used, with a spray bar containing Teejet^®^ XR 11002VS nozzles (TeeJet Technologies, Springfield, IL, USA), spaced 0.5 m from each other at a height of 0.5 m of the target and calibrated at a constant pressure of 200 kPa to obtain an average spray volume equivalent to 200 L ha^−1^ (~0.5 mL per pot). The per-hectare doses were converted to a fixed amount of herbicide per pot, considering the recommended field rate and the standardized spray volume per plant. This ensured that each experimental unit received the correct proportional dose. At the time of application, each specific treatment was removed from the greenhouse separately, in order to avoid contamination from the other treatments. Environmental conditions at the time of application for plants at the 4–6 leaf and 1–3 tiller stages were as follows, respectively: relative air humidity of 78 and 75%, air temperature of 22.3 and 23.5 °C, and wind speed of 2 and 3 km h^−1^.

The specific leaf area (SLA), the difference between the temperature of the environment at the moment of application and the leaf temperature, and the stomatal conductance (gs) were determined on the same day and before the application of the different doses of glyphosate, to verify the physiological and morphoanatomical alterations according to the types of water management studied.

The specific leaf areas of the plants in each water management treatment were measured with a leaf area meter, the LI-3100C Area Meter (LI-COR^®^ Environmental, Lincoln, NE, USA). For this evaluation, one leaf of each plant was collected and measured. Posteriorly, the leaves collected were taken to a forced-air ventilation oven at 65 °C until reaching a constant mass to obtain each treatment’s leaf dry matter. Based on these values, the following formula was used to determine SLA (Equation (2)):

(2) $$\mathrm{SLA}\;=\;\frac{\mathrm{LA}}{LDM}$$

where:

LA = leaf area of each experimental unit;
LDM = leaf dry matter of the unit.

Stomatal conductance (gs) and leaf temperature were evaluated in the adaxial surface with the Leaf Porometer Model SC-1 (Decagon Devices, Meter Group, Pullman, WA, USA). Based on the values of the leaf and environment temperatures, it was possible to determine the difference between the environment temperature and the leaf temperature at the moment of evaluation.

The control of crabgrass was visually evaluated 7 and 14 days after the application (DAA) through grade percentage scales, where zero represents a total absence of injuries and 100 represents plant death [8]. At 35 DAA, the shoot and the root system of the plants were collected, washed, and posteriorly maintained in a forced-air ventilation oven at 65 °C until reaching a constant weight to determine shoot dry matter and root dry matter (g).

### 2.4. Statistical Analysis

The results obtained for the control percentage, shoot dry matter, and root system dry matter of crabgrass were submitted to analysis of variance by the F test at *p* ≤ 0.05. The means were compared by Student’s *t*-test (*p* ≤ 0.05).

The physiological variables, such as the SLA, the difference between the environment temperature and the leaf temperature, and the gs, were analyzed through the confidence interval, with a confidence coefficient of 95% for the averages (µ), but were not subjected to a means comparison test. For this parameter, the following formula was used (Equation (3)):

(3) $${\mathrm{IC}\left(\mu \right)}_{95\%\;}=\;\mu \;\pm \;t\;\frac{s}{\sqrt{n}}$$

where:

μ = repetition average;
t = tabulated t value;
s = standard deviation;
n = number of samples.

All statistical analyses were conducted with the statistical program AgroEstat version 1.1.0.712 [9].

## 3. Results

**Figure 1:**
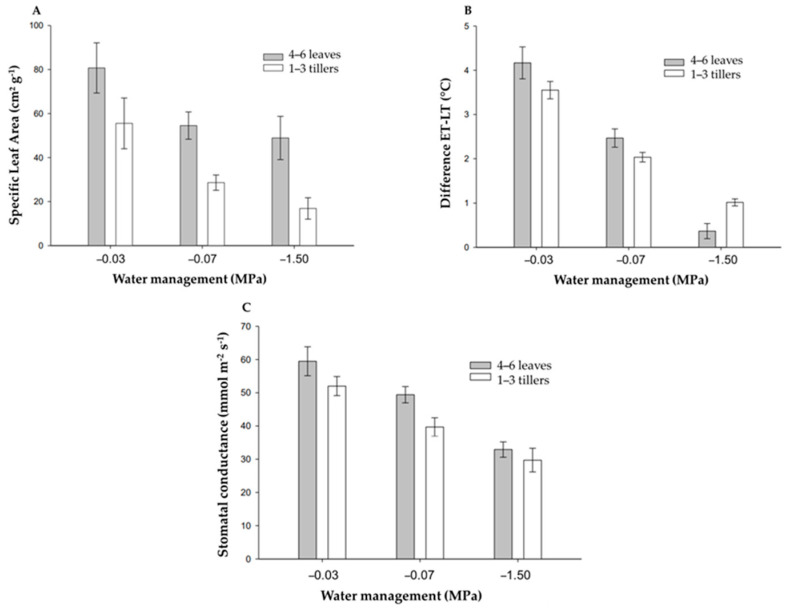
Specific leaf area (SLA) (**A**); differences between environmental temperature (ET) and leaf temperature (LT) (**B**), and the stomatal conductance (gs) (**C**) of crabgrass plants in the stages of 4–6 leaves and 1–3 tillers, submitted to different water management of the soil before applying the herbicide glyphosate doses. Note: The bars in each mean represent the confidence interval, with a confidence coefficient of 95% for the averages.

Crabgrass plants grown in soils with different water availability differed for the SLA variable in both studied developmental stages (4–6 leaves and 1–3 tillers). The plants exhibited the highest specific leaf area (SLA) under greater soil water availability (−0.03 MPa) compared with the −0.07 and −1.5 MPa treatments, at both the 4–6 leaf and 1–3 tiller developmental stages. It is essential to highlight that *D. nuda* subjected to the water regimes of −0.07 and −1.5 MPa at the stage of 4–6 leaves showed mean SLA values similar to the confidence interval. It is noted that regardless of the water management used, plants in the 4–6 leaf development stage presented the highest SLA values compared to plants in the 1–3 tiller development stage (Figure 1A).

The water management to which the crabgrass plants were subjected significantly influenced their leaf temperature. The plants with the lowest water availability (−0.07 and −1.5 MPa) had the highest leaf temperatures, which means the lowest values for the variable difference between the environmental temperature and the leaf temperature, regardless of the evaluated development stage (Figure 1B).

A similar pattern of stomatal conductance (gs) was observed in crabgrass across the different soil water conditions at both the 4–6 leaf and 1–3 tiller stages. Plants grown without water restriction exhibited significantly higher mean gs values than those under the other water management regimes. However, it is noteworthy that the more developed crabgrass plants (1–3 tillers) apparently had lower gs values under optimal development conditions (−0.03 MPa), when compared to the same management method at the stage of 4–6 leaves (Figure 1C).

Based on the analysis of variance of the mean percentage of crabgrass control under different water management regimes and glyphosate doses applied at distinct developmental stages, all main factors and their interactions were significant at 7 days after application (DAA). At 14 DAA, no significant main effects were observed; significance was detected only for the two-way interaction between developmental stage and water management and for the three-way interaction among developmental stage, water management, and glyphosate dose (Table 2).

When comparing the interaction between “water management” and “development stage” at 7 DAA, crabgrass plants in the early stages of development (4–6 leaves) were more affected by glyphosate dose applications compared to the plants controlled at a later development stage (1–3 tillers), regardless of water management. Higher water availability (−0.03 MPa) provided the greatest control in both development stages (Table 3).

In the interaction between development stage and glyphosate dose, also at 7 DAA, greater control efficiency was observed in plants with 4–6 leaves, with differences of approximately 45.5 and 40.0% at glyphosate doses of 270 and 540 g ha^−1^, respectively. The glyphosate dose representing 50% of the one recommended by the manufacturer (270 g ha^−1^) was similar to the dose value representing 100% of the recommendation when the herbicide was applied to younger plants (4–6 leaves). Greater efficiency of glyphosate was observed when applied to plants grown without water restriction, regardless of dose. Under a high water deficit, the application of 270 g ha^−1^ resulted in reduced control at 7 DAA (Table 3).

At 14 DAA, lower herbicide action was observed under −0.03 MPa when 270 g ha^−1^ was applied. The control of crabgrass plants under −0.07 and −1.5 MPa was similar, and the application of 540 g ha^−1^ provided adequate control regardless of water management. Reduced control was observed when the herbicide was applied at later stages, with 540 g ha^−1^ being the most efficient dose at the 1–3 tiller stage. At the 4–6 leaf stage, both 270 and 540 g ha^−1^ resulted in approximately 100% control by 14 DAA, whereas at the 1–3 tiller stage this occurred only at 21 DAA (Table 4).

At 35 days after application, shoot dry matter was significantly affected only by water management and glyphosate dose, either as main effects or through their interaction. A significant interaction between water management, glyphosate dose, and development stage was observed for root dry matter (Table 5).

The highest shoot dry matter accumulation occurred under full water availability (−0.03 MPa), regardless of glyphosate dose. Reductions of 78.48 and 75.94% were observed with glyphosate doses of 270 and 540 g ha^−1^, respectively. Under a water deficit, shoot dry matter accumulation was reduced regardless of glyphosate application, with reductions of 56.96 and 82.27% at −0.07 and −1.5 MPa, respectively (Table 6).

Glyphosate application reduced root dry matter accumulation in both development stages. Reductions of 71.79 and 74.35% were observed at 270 and 540 g ha^−1^ when applied at the 4–6 leaf stage, while reductions of 92.30 and 86.15% were observed at the 1–3 tiller stage. Root dry matter accumulation was 40% lower when plants were controlled at the 4–6 leaf stage compared to the 1–3 tiller stage (Table 7).

Regardless of development stage, the treatment without water restriction promoted the greatest root dry matter accumulation, especially when control occurred at the 1–3 tiller stage. Root dry matter accumulation decreased with reduced water availability in the absence of glyphosate, with reductions of 59.40% at −0.07 MPa and 86.13% at −1.5 MPa. Under a severe water deficit, root dry matter accumulation was not influenced by glyphosate application (Table 7).

## 4. Discussion

The reduction in SLA under water-restricted environments indicates decreased leaf blade dimensions, which may be related to reduced cell expansion caused by decreased turgor pressure and reduced water pressure on the cell wall [10]. Higher SLA values in early developmental stages indicate lower accumulation of leaf dry matter relative to leaf area. Specific leaf area is correlated with physiological and chemical variables, and low SLA values may indicate higher concentrations of cytoplasmic components, influencing dry matter accumulation and maintaining lower SLA in later stages [11]. These morphological adjustments may represent adaptive strategies that allow plants to maintain metabolism under stressful conditions [12,13].

The leaf temperature differences observed under a water deficit confirm the relationship between leaf temperature and plant water status. Under adequate water availability, transpiration cools leaves, whereas under a water deficit, transpiration decreases, increasing the leaf temperature [14,15]. This response is closely related to stomatal conductance, as increased stomatal resistance reduces transpiration, the primary mechanism of heat dissipation in plants [16].

Reduced gs in more developed plants under optimal water conditions suggests an age-related reduction in the stomatal opening as a mechanism to limit water loss. A water deficit stimulates ABA accumulation in guard cells, reducing stomatal conductance [6]. Variations in stomatal number and size under water stress further contribute to reduced water loss and influence CO_2_ uptake for photosynthesis and dry matter accumulation [17]. Reduced gs and transpiration under water restriction may also affect energy balance and water use efficiency in monocotyledonous plants [7,16].

Greater glyphosate efficiency in younger plants and under higher water availability conditions may be related to increased herbicide absorption and translocation. Under severe water restriction, thicker cuticles may limit glyphosate penetration, as absorption depends on leaf hydration and cuticle permeability [5,18,19]. Higher metabolic activity and stomatal conductance in younger plants may further enhance herbicide efficacy (Figure 1C).

Reduced control efficiency in later developmental stages may be associated with increased tissue differentiation and cuticle maturity, which can reduce herbicide absorption and translocation [20,21,22]. Although older plants have a greater leaf area, anatomical and architectural characteristics may limit rapid control [22,23].

A water deficit significantly reduces shoot and root dry matter accumulation, reflecting the trade-off between water conservation and CO_2_ assimilation under stress conditions. Stomatal closure under a water deficit limits growth and development [16]. Reduced root dry matter accumulation under glyphosate application varied with the development stage, indicating that earlier control limits biomass accumulation by shortening the growth period [5].

The lack of a glyphosate effect on root dry matter under a severe water deficit is consistent with observations in other species, such as *Urochloa decumbens*, under −1.5 MPa [7]. Under high stress, plants may allocate assimilates preferentially to root formation [24,25]. ABA accumulation under a water deficit affects both shoot and root development, contributing to reduced stomatal conductance and altered morphophysiological responses [6]. Roots exhibit greater tolerance to reduced water potential than shoots due to more efficient osmotic adjustment and slower turgor loss, making root growth less sensitive to osmotic stress [26,27].

## 5. Conclusions

With increasing water restriction, there is a decrease in the specific leaf area, stomatal conductance, the difference between environmental and leaf temperature, and in the shoot and root dry matter accumulations in crabgrass, regardless of the development stage. The development stage of 4–6 leaves of crabgrass plants influences the control provided by the herbicide glyphosate, irrespective of the dose used. The different water deficits studied reduce the power of the herbicide glyphosate in the two crabgrass plants’ development stages. Thus, it is recommended to prioritize glyphosate application during periods of adequate soil water availability or after rainfall events.

## Figures and Tables

**Table 1:** Relation between potentials (MPa) and contents (%) of soil water used in this study.

Retained Water (dm^3^ dm^−3^)						
Tension (MPa)						
Saturated	−0.01	−0.03	−0.05	−0.07	−0.5	−1.5
39%	14%	13%	11%	10%	9%	8%

**Table 2:** Analysis of variance of the percentage of crabgrass control following application of different glyphosate doses under distinct water management regimes and at different developmental stages.

Variation Factor	Days After Application	
	7	14
Development stage (S)	755.9 **	71.30 **
Water management (M)	45.5 **	4.79 *
Glyphosate doses (D)	2600.1 **	65,051.2 **
S × M	5.55 **	1.51 ^ns^
S × D	190.8 **	18.70 **
M × D	17.8 **	3.16 *
S × M × D	11.2 **	0.76 ^ns^
CV%	8.3	1.7

ns, * and ** indicate non-significant results, and significance at *p* ≤ 0.05 and *p* ≤ 0.01, respectively, according to the F test.

**Table 3:** Mean percentages of crabgrass control at seven days after application of different glyphosate doses, according to the developmental stage at the time of application and the water management regime.

Water Management (MPa)	Development Stage			
	4–6 Leaves	1–3 Tillers		F_Stage_
−0.03	56.8 aA	37.8 aB		173.4 **
−0.07	50.2 bA	25.1 cB		304.8 **
−1.5	52.0 bA	29.6 bB		288.8 **
F_Management_	10.6 **	40.5 **		
**Doses** **(g ha^−1^)**	**Development Stage**			
	**4–6 Leaves**	**1–3 Tillers**		**F_Stage_**
0	0.0 bA	0.0 cA		-
270	79.5 aA	43.3 bB		633.7 **
540	81.4 aA	49.1 aB		503.9 **
F_Dose_	2091.8 **	699.1 **		
**Doses** **(g ha^−1^)**	**Water Management (MPa)**			
	**−0.03**	**−0.07**	**−1.5**	**F_Management_**
0	0.0 bA	0.0 bA	0.0 cA	-
270	69.9 aA	57.1 aB	57.3 bB	34.6 **
540	72.0 aA	55.8 aC	68.1 aB	46.5 **
F_Dose_	1084.3 **	686.2 **	865.3 **	

Means followed by the same lowercase letter in the column and uppercase letter in the line do not differ by the “*t*” test (*p* ≤ 0.05). ** indicate significant results at *p* ≤ 0.01, according to the F test.

**Table 4:** Mean percentages of crabgrass control at 14 days after application of different glyphosate doses, according to the water management regime and the developmental stage at the time of application.

**Doses** **(g ha^−1^)**	**Water Management (MPa)**			
	**−0.03**	**−0.07**	**−1.5**	**F_Management_**
0	0.0 cA	0.0 bA	0.0 bA	-
270	96.4 bB	98.9 aA	98.0 aA	10.9 **
540	98.4 aA	98.8 aA	98.6 aA	0.3 ^ns^
F_Dose_	21,341.0 **	21,968.8 **	21,747.7 **	
**Doses** **(g ha^−1^)**	**Development Stage**			
	**4–6 Leaves**	**1–3 Tillers**		**F_Stage_**
0	0.0 bA	0.0 cA		-
270	99.5 aA	95.9 bB		68.1 **
540	100.0 aA	97.2 aB		40.6 **
F_Dose_	33,610.0 **	31,460.0 **		

Means followed by the same lowercase letter in the column and uppercase letter in the line do not differ by the “*t*” test (*p* > 0.05). ns and ** indicate non-significant results, and significance at *p* ≤ 0.01, respectively, according to the F test.

**Table 5:** Analysis of variance of shoot dry matter and root dry matter of crabgrass plants after applying different glyphosate doses according to the water management and development stage at the time of application.

Variation Factor	Dry Matter	
	Shoot	Root
Development Stage (S)	3.43 ^ns^	13.59 **
Water Management (M)	98.18 **	95.13 **
Glyphosate Dose (D)	142.34 **	254.52 **
S × M	1.08 ^ns^	5.27 **
S × D	2.17 ^ns^	29.97 **
M × D	37.78 **	86.59 **
S × M × D	1.8 ^ns^	4.78 **
CV%	33.42	32.68

ns and ** indicate non-significant results, and significance at *p* ≤ 0.01, respectively, according to the F test.

**Table 6:** Shoot dry matter accumulation (g) of crabgrass at 35 days after application of different glyphosate doses under distinct water management regimes.

Doses (g ha^−1^)	Water Management (MPa)			
	−0.03	−0.07	−1.5	F_Management_
0	0.79 aA	0.34 aB	0.14 aC	163.34 **
270	0.17 bA	0.09 bB	0.08 abB	3.2 *
540	0.19 bA	0.07 bB	0.06 bB	7.19 **
F_Dose_	182.94 **	32.76 **	2.02 ^ns^	

Means followed by the same lowercase letter in the column and uppercase letter in the row do not differ by the “*t*” test (*p* ≤ 0.05). ns, * and ** indicate non-significant results, and significance at *p* ≤ 0.05 and *p* ≤ 0.01, respectively, according to the F test.

**Table 7:** Crabgrass root dry matter accumulation (g) at 35 days, after applying different glyphosate doses according to the development stage at the time of application and water management.

**Development** **Stage**	**Doses (g ha^−1^)**			
	**0**	**270**	**540**	**F_Dose_**
4–6 leaves	0.39 bA	0.11 aB	0.1 aB	55.47 **
1–3 tillers	0.65 aA	0.05 aB	0.09 aB	229.01 **
F_Stage_	70.46 **	2.98 ^ns^	0.08 ^ns^	
**Development Stage**	**Water Management (MPa)**			
	**−0.03**	**−0.07**	**−1.5**	**F_Management_**
4–6 leaves	0.37 bA	0.12 bB	0.11 aB	44.01 **
1–3 tillers	0.44 aA	0.26 aB	0.10 aC	56.39 **
F_Stage_	4.66 *	19.44 **	0.03 ^ns^	
**Doses** **(g ha^−1^)**	**Water Management (MPa)**			
	**−0.03**	**−0.07**	**−1.5**	**F_Management_**
0	1.01 aA	0.41 aB	0.14 aC	266.94 **
270	0.07 bA	0.09 bA	0.09 aA	0.12 ^ns^
540	0.12 bA	0.06 bA	0.09 aA	1.25 ^ns^
F_Dose_	376.25 **	50.01 **	1.43 ^ns^	

Means followed by the same lowercase letter in the column and uppercase letter in the row do not differ by the “*t*” test (*p* ≤ 0.05). ns, * and ** indicate non-significant results, and significance at *p* ≤ 0.05 and *p* ≤ 0.01, respectively, according to the F test.

## Data Availability

The data supporting this study’s findings are available from the corresponding author upon reasonable request.
